# Emergence of Leadership in a Group of Autonomous Robots

**DOI:** 10.1371/journal.pone.0137234

**Published:** 2015-09-04

**Authors:** Francesco Pugliese, Alberto Acerbi, Davide Marocco

**Affiliations:** 1 Department of National Accounts and Economic Statistics, Italian National Institute of Statistics, Rome, Italy; 2 Department of Archaeology and Anthropology, University of Bristol, Bristol, United Kingdom; 3 Philosophy & Ethics, School of Innovation Sciences, Eindhoven University of Technology, Eindhoven, The Netherlands; 4 School of Computing and Mathematics, University of Plymouth, Plymouth, United Kingdom; Peking University, CHINA

## Abstract

In this paper we examine the factors contributing to the emergence of leadership in a group, and we explore the relationship between the role of the leader and the behavioural capabilities of other individuals. We use a simulation technique where a group of foraging robots must coordinate to choose between two identical food zones in order to forage collectively. Behavioural and quantitative analysis indicate that a form of leadership emerges, and that groups with a leader are more effective than groups without. Moreover, we show that the most skilled individuals in a group tend to be the ones that assume a leadership role, supporting biological findings. Further analysis reveals the emergence of different “styles” of leadership (active and passive).

## Introduction

Many animal species, including humans, live in groups. Grouping provides members with several benefits, including (a) mating [1], (b) reproducing [2], (c) caring of offspring [3], (d) protection from predators [4], (e) feeding efficiency [5], (f) supporting competition with other groups of con-specifics [6], (g) information sharing [7]. On the other hand, the most significant disadvantages of grouping are: a) intra-group competition [8]; b) coordination needs [9]. Point (b) implies a negotiation problem, often not easy to solve [10]. Ultimately, living in groups poses a fundamental problem of social coordination. Many have argued that the emergence of leadership-followership patterns (over years of evolution) is indeed a way to solve this coordination problem [11]. The term leadership indicates, in this context, any individual behaviour that influences the type, timing and duration of the whole group activity [12]. Across species, individuals are more likely to emerge as leaders if they have particular morphological, physiological, or behavioural traits increasing their propensity to act first in coordination problems [13]. Correlations between leadership and temperaments are well documented in animal and human literature [14]. In a recent experiment, pairs of sticklebacks had to coordinate their foraging toward a food patch and personality differences seemed to play a crucial role for achieving coordination [15]. Bold fish emerged as leaders and shy fish emerged as followers. In humans, extroversion is correlated with leadership, and this trait (an indication of boldness) has a substantial heritable component [16]. Furthermore, experiments show that the most talkative members of a group very often assume the role of leader, more or less regardless of the quality of their inputs (referred to as the “babble effect”, [17]). Game-theoretical analysis has also explored leadership emergence [18]. In a simple two-player “coordination game”, two individuals are required to move together and, in the same time, to seek resources. In this situation, any trait (physical or behavioural) that strengthens the likelihood of one individual to move first will make him more likely to emerge as the leader [19]. The consistent correlation between leadership and personality variability suggests the possibility that differences of personality are maintained in populations because they foster the emergence of leaders and followers [20]. A new study proves that in macaques there is a strong correlation between social-ranking and brains’ morphologies of the individuals [21]. Spider monkeys distribute themselves in groups with a strong component of leadership in order to reach food patches, thus for social coordination [22]. An ample literature exists about the emergence of leadership for group coordination in insects social systems. Group-living insects are often faced with the problem of choosing between one or more alternative resource sites. A central question in such collective decision-making includes determining which individuals induce the decision and when. For instance, every year, faced with the life-or-death problem of choosing and traveling to a new nest, honeybees deal with the election of a leader [23]. The experimental studies about shelter selection by cockroach groups demonstrates that choices can emerge through strategies of distributed leadership. This mechanism leads to optimal mean benefit for group individuals [24]. In ants, leaders perform chemical modulations in order to drive the colony to select the most profitable food source among several ones. Some researchers proved how a minority of ants can influence the social system through a distributed leadership [25]. In this work we intend to investigate the specific problem of the leadership emergence for the nest selection in insects by modeling it with an alternative and original approach grounded on “Evolutionary Robotics” [26]. In the past, some researchers already used artificial agents and robots to investigate the emergence of leadership. However, such research relied on homogeneous individuals [27]. On the contrary, we believe a variation of individuals “personalities” must be kept, according to the literature we previously mentioned. To this end, we designed a possible variation of the Genetic Algorithm [28] that we called “Heterogeneous Genetic Algorithm” (HGA). HGA enables us to sustain genetic variations among a colony of autonomous robots, by splitting them into different populations which evolve and are ranked separately. Our experiments have a two-pronged value. In robotics and software design, the genetic differentiation of robots control systems might lead to the development of a new generation of autonomous robots for which high levels of coordination are critical and a spontaneous occurrence of leadership is the only manner to reach such a coordination. Many efforts in military research have been dedicated in the direction of collective robotics, with the aim of developing a network of cooperative autonomous robots which work together to search, track, carry, deploy and retrieve sensors and other small payloads for a variety of purposes [29]. The industry of mobile sensors investigated the navigational capabilities of a swarm of mobile sensors or robots for maximizing local and global tasks such as firefighting, landmine detection, radioactivity detection, etc. Namely, the navigation task in this case is aimed at locating desirable target sources in a given sensing area [30]. Finally, recent advances in nano-technology led to nano-robots, which are effectively used as nano-medicine. Future medical research will deal with the issue of injecting nano-robots into the human body to perform treatments on a cellular level. Nano-robots placed inside the human body will encounter the immune system as an obstacle during their flowing within a human body. Thus the group of nano-robots must use a strategy to navigate and avoid such immune system collectively [31]. Our experiments may give insights in such domains. In military research, for example, where groups of autonomous and cooperative robots might easily assume leader/follower patterns in order to coordinate for the tasks of search, track or carry commodities to a target area. Mobile sensors or robots may implement leadership strategies for groups containing multiple units in order to optimise group tasks of localisation like radioactivity detection, landmine detection, etc. In medical nano-robotics, to avoid obstacles trajectory such as immune system cells, a self-organised trajectory planning is required. Leadership strategies might lead to a swarm optimisation by a self-organised and bio-inspired control of nano-scale robots fostering the obstacle avoidance throughout the trajectory of the nano-robots group movements. On the other hand, in social science, our results may originally contribute to address some important questions such as: is leadership unavoidable for a social decision-making problem? Who is the leader? What are leaders made of? What are the characteristics and skills of a leader? The central idea is whether, as in biological literature, individual behavioural differences play a fundamental role. At the same time, we study the emergence of different types of leadership, such as passive and active leadership [32], that are aspects concerning leadership not much documented in biology. Passive leadership usually occurs when individuals possess pertinent information and there is no need to actively communicate this knowledge to group-mates to assume leadership roles. In this case they may simply apply heuristics such as “adopt the same direction as those that are close by” or “avoid becoming isolated”. Such passive leadership is most common in large and homogenous insect swarms where individuals have no significant conflict of interest [33]. On the other hand, active leadership occurs when potential leaders explicitly signal their intention to other group members that can choose whether to follow or not [34, 35]. As an example, in ants (Temnothorax albipennis) individuals who have learnt the route to feeding sites use “tandem running” to lead another ant from the nest to food, by means of signals between the pair of ants controlling both the speed and course of the run [36]. In migrating honeybee colonies, leaders actively play a role in a two-part process that involves deciding where to go, and then guiding the swarm to the selected site. Specifically, lead individuals (scouts) recruit followers using “dances” that inform proximate colony members on the location of new nest sites [37].

## Experimental Setup

### The Environment and the robot’s perceptive system

**Fig 1:**
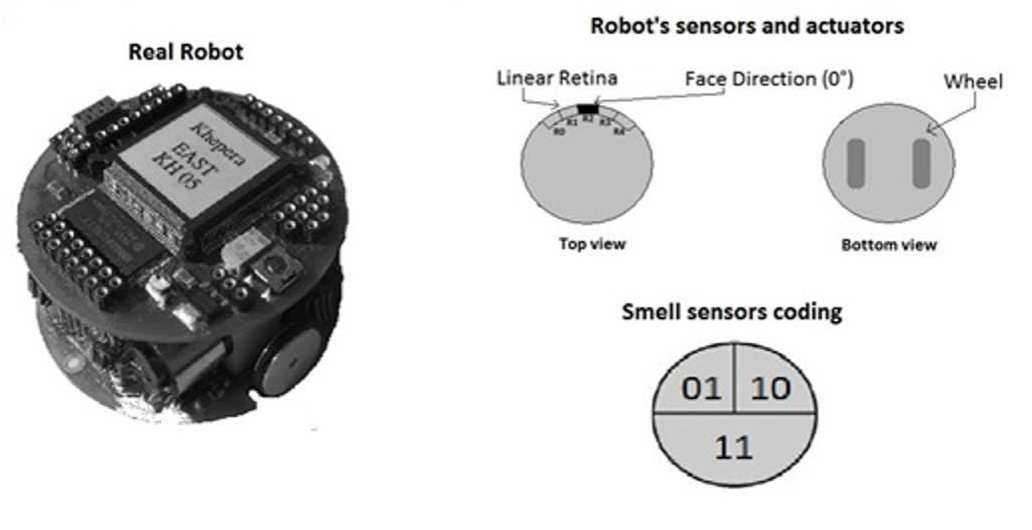
Schematisation of top and bottom view of the robot chassis.

For the experiments, we simulated four real robots called Khepera [38]. Every Khepera robot is a small differential wheeled mobile robot made of a circular chassis with a diameter measuring 5.5 cm. This type of robots was originally developed at the LAMI laboratory at EPFL (Lausanne, Switzerland) in the mid ‘90s for research purposes. Later on, the Khepera helped in the emergence of Evolutionary Robotics (ER). ER is an approach that makes use of evolutionary computation in order to develop controllers (i.e. neural networks) for autonomous robots. One of the algorithms used in evolutionary computation are the Genetic Algorithms (GAs) where a population of candidate controllers is continuously optimised by means of a set of “genetic” operators (i.e. mutation) according to a fitness function (the whole process will be detailed in the section “Artificial Evolution”). Since the evolution of controllers is an onerous activity (from a computational standpoint), ER methodology usually exploits simulations as much as possible. It is common that today’s machine learning methodologies make more use of simulation to develop control systems of autonomous robots as the training (or evolution) of the slow robots’ micro-controllers discourages researchers to use physical robots during the training period. By evolving neural controllers for a Khepera robot in computer simulations and then transferring the agents to the real world, some researcher showed that an accurate model of a particular robot-environment dynamics can allow to get the same results once agents’ neural networks are transferred on real robots [39, 40]. Experimental environment is a squared arena surrounded by walls of 110 cm length, populated by a group of four Khepera Robots. Every time robots impact one another or the walls, these are repositioned in nearby location choosing a random new orientation. The arena’s floor is white and contains two circular grey areas with a diameter of 22 cm each. These areas represent the food sources and are placed at two fixed locations within the environment. The Robot bodies are equipped with two motors controlling the movements of two wheels respectively. The four robots’ cases are marked by distinctive colours: green, blue, light-blue or yellow. The bottom of the Khepera Robot is already provided with a ground sensor which enables the robot to perceive the ground colour tones underneath the robot. We exploited this feature to detect the food zones drawn on the arena’s ground once the robot is positioned on the top of it. However, from the outset, we needed our robots to be able to locate food zones from afar. This because robots have no other means to orientate in order to reach food zones within the environment. Thus we imagined a new kind of sensors for the simulated robots that we called “Smell Sensors”. Smell sensors are able to identify the presence of a food zone from a certain distance. They are activated according to which robot’s quadrant is facing to the food zone in a given moment. Smell sensors are 3 and have been placed in precise angles of the robot’s chassis. The first, placed at the degree 45° from the face direction (0°), manages an angle interval between 0° and 90°. The second, located at 180° from the face direction, is able to manage an angle from 90° to 270°. The third smell sensor is placed at the angle 315° and manages the interval from 270° to 360°. So when one of the 3 quadrants faces a food zone, the related smell sensor will be activated from any distance within the arena, as the range of smell sensors covers the entire environment. Smell sensors are activated with two digits of binary code encoding the robot’s quadrant that is facing a food zone in a given moment. See Fig 1 for a full depiction of the real robot and the sensors/actuators settings.

**Fig 2:**
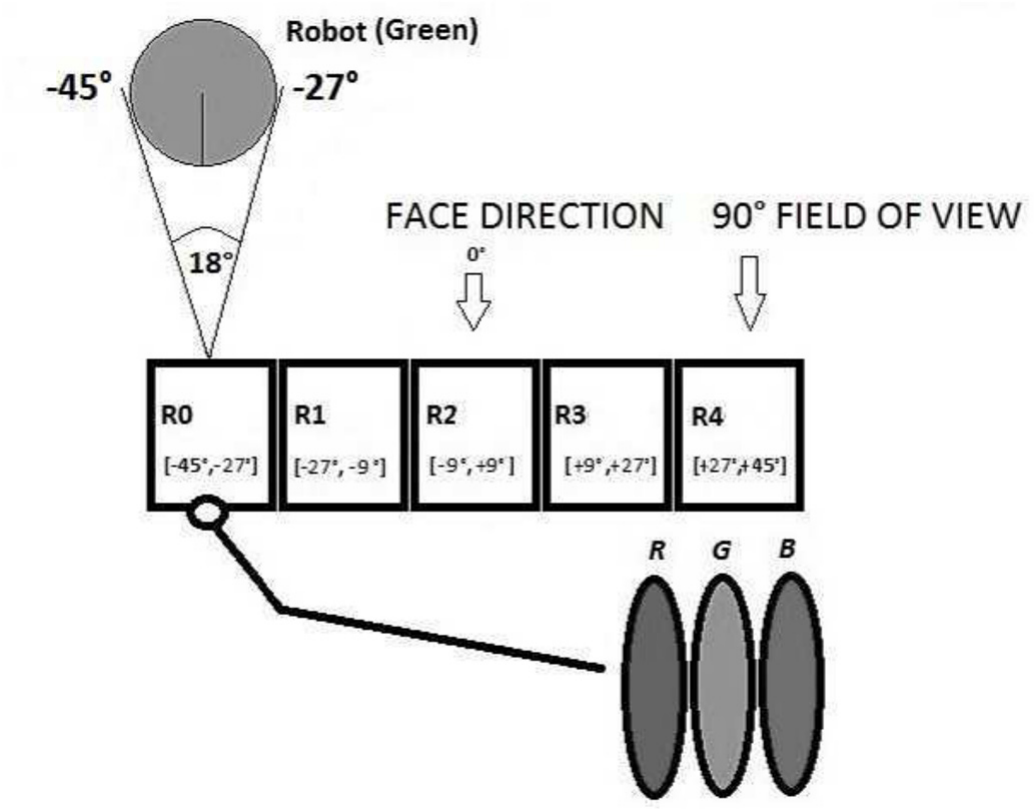
A schematisation of Robot’s Vision Systems.

The front of the robot is provided with a linear retina in order to make it capable of detecting the relative position and the colour of other robots. The linear retina is composed of five RGB photo-receptors that manage a specific portion of each robot’s field of view (FOV). The range of view is 90 degrees wide and expresses the extent of the observable world for each robot. The FOV (see Fig 2) ranges from -45 degrees to +45 degrees with regard to the direction (0°) being faced.

In this manner, each photoreceptor operates on an 18 degree wide portion of the FOV, the first one is associated with the range [-45°, -27°] in respect to the direction of motion, the second one is associated with [-27°, -9°], and so on. Each photoreceptor consists of 3 photosensitive components, respectively Red, Green, and Blue. When an object is located in the FOV, the photo-receptors involved are activated by the colour of that object. The maximum vision distance of the receptors is the size of the entire environment. The structure of the linear retina makes the Khepera robots not able to possess information about the size of the other robots as they have no means to infer this. They can only measure other robots size in a relative way: for instance if the size of the green spot increases on their retina’s photo-receptors this means they are getting closer to the green robot and if the spot is getting smaller they are moving away from the green robot. Given the sensory system, robots are neither able to measure other robots sizes nor to locate their absolute positions. They can just understand whether they are getting closer or further from one another robot. Finally, each robot is not able to understand its absolute position within the environment but only its relative position with respect to the food zones.

### Nervous System

**Fig 3:**
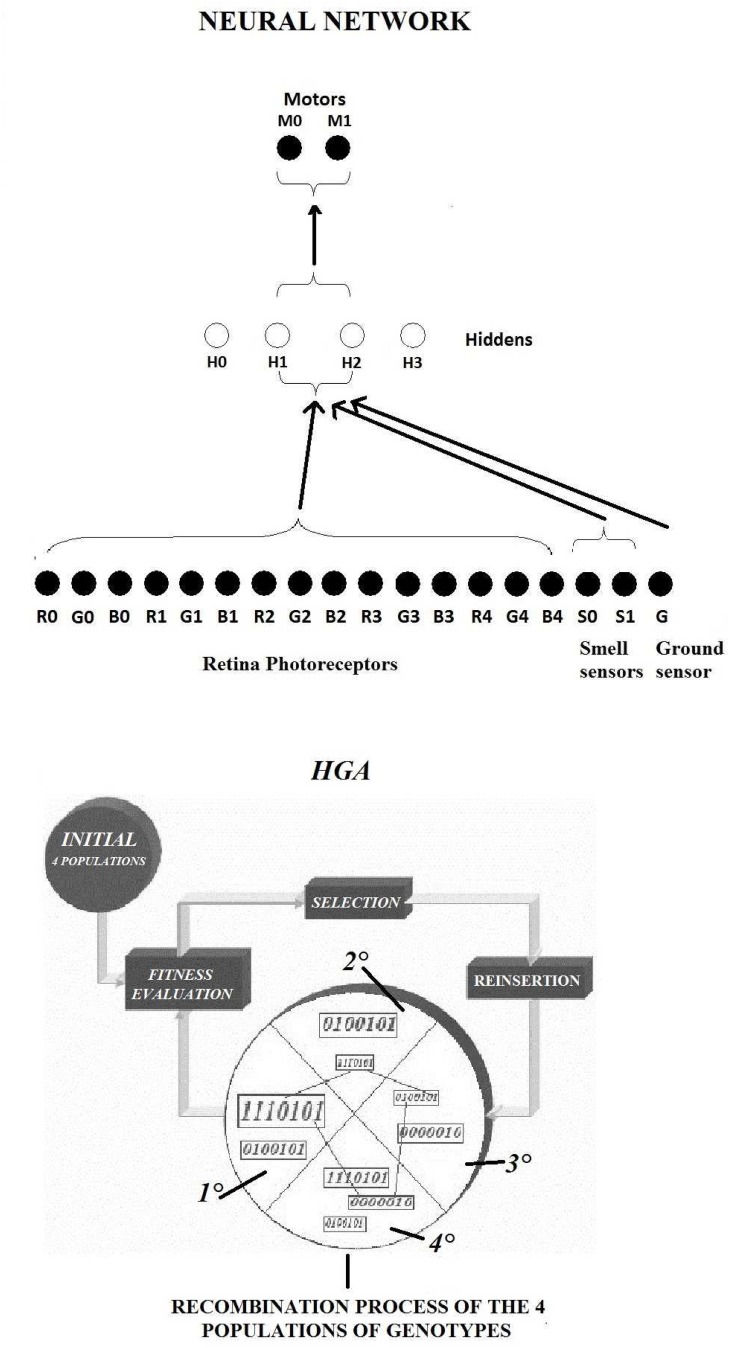
Neural network architecture and the Heterogeneous Genetic Algorithm.

The Robots’ control system consists of a feed-forward neural network made of 24 neurons which are distributed on 3 layers: input, hidden and output. Each layer is linked to the next one by a pattern of synaptic connections. Input layer includes 15 neurons (from R0,G0,B0 to R4,G4,B4) encoding the activation state of the corresponding 15 RGB photo-receptors components. The RGB signals ranging within [0, 255] are pre-normalised into a range of signals [0, 1] appropriate to the input of the neurons. Another 2 input neurons (S0, S1) encode the activation from the binary smell sensors. Lastly, 1 neuron (G) encodes the data from the ground sensor which generates a signal between 0 and 1 according to the floor’s colour shade. The hidden layer consists of 4 neurons. The output layer is made of 2 neurons controlling the speed of two motorised wheels, respectively. The neural network’s topology is depicted in Fig 3.

### Artificial evolution

According to the Evolutionary Robotics, all the neural controllers of the robots undergo an evolutionary process based on a ranking-type Genetic Algorithm. The free parameters of each neural network, namely the synaptic weights of connections and biases are encoded into the individual genotypes upon which the genetic algorithm operates. Connection weights are scaled into the range [-5.0, +5.0] and encoded into 8 bit sequences. The idea behind the “Heterogeneous Genetic Algorithm” (HGA) is that each robot in the team belongs to a different population of 20 individuals. The entire evolutionary process occurs through 600 generations and starts from 4 populations of totally “naive” robots, where each initial population consists of random genotypes. Therefore, at the generation 0, robots are endowed with no skills and no proper attitude about how to move and identify the food sources. At each generation, 1 genotype is randomly extracted from each population and loaded into one robot’s controller. The 4 robots produced by this extraction process are left to act in the environment for all the life-time, lasting 3000 time steps. Then the behaviour of each robot is evaluated by means of a fitness function. This process is replicated for 20 trials where a sequence of different indexes is randomly generated for each trial. For instance, the sequence 3-4-5-11 means that the genotype no.3, from the first population, controls the green robot, the genotype no.4, from the second population, controls the blue robot, the genotype no.5 from the third population controls the light-blue robot, and the genotype no.11 manages the yellow robot. The same index sequence will never be extracted twice in the same generation. The modality of this sequences recombination is schematised in Fig 3. Fitness is explicitly designed to foster cooperative/coordinated behaviours: individuals get a score of +1.0 for each time step the whole group is located within the same food zone. Individuals receive 0.0, otherwise. It is worth nothing here that before resorting to the fitness function described above, we made multiple tests with different fitness functions. In a first scenario we considered exclusively an individual fitness. Namely, individuals gain +1.0 for each time step they are positioned on the top of a food zone, they receive 0.0, otherwise. In this case robots tend to maximize their individual feeding without any care of the others status and no leadership emerged because. In a second scenario a collective fitness measure was added to the individual one. Although this might seem a more plausible scenario, and many replications showed leadership emergence, this scenario posed a number of measuring issues, as it was difficult to establish the fitness component capable of influencing the overall behaviour. Instead, using exclusively a collective fitness, higher levels of group fitness always correlate to higher levels of social coordination, because they occur when robots reach the food zone together. At the end of 20 trials, and so at the end of one single generation, each population is separately ranked according to the fitness score. For each population, the 4 highest-ranked individuals are selected from the list of genotypes for each population. Each of the best individual generates 5 offspring which inherit the father’s genotype. The first offspring wholly preserves the genotype of the father (elitism) while the other four undergo a random mutation by a likelihood of 2% (mutation rate). As mentioned above, we explicitly reward cooperation, since each population evolves separately, the mechanism facilitates, in the same time, the genetic differentiation amongst the group’s members and allows the robots to evolve their behavioural skills distinctly. The evolutionary process has been replicated 30 times with a different initial random population. All the evolutionary process runs on the simulation environment on a server. Evolutionary computation requires high workloads and it cannot be executed on the slow and simple CPUs of real wheeled robots. That is why the only way to evolve robots controllers is in simulation environments where a very powerful computer can support high computational loads. Once the simulated robots get smarter, they can be “transferred” onto the real robots for testing. Therefore all the computational load for the evolutionary process and testing of the simulated robots is executed on the server.

## Methods

We have approached to the analysis of the results by using two different standpoints: a behavioural analysis (qualitative) and a numerical analysis (quantitative). The behavioural analysis consists in a qualitative description of the robotic group’s behavioural dynamics. This analysis has been performed by watching the last generation’s best team of each replication. The numerical analysis consists in identifying some indicators able to quantitatively describe specific properties of teams such as leadership. In this regard, we could not found any suitable leadership measure from the biological literature, despite a long investigation, and we concluded that we needed to devise new and simple behavioural indicators, which are explained below.

### Does Leadership emerge?

To reveal whether leadership arises in the group we measure the average distance between each group’s members and the barycenter of the group itself (“Barycenter measure”). The underpinning hypothesis is that the leader robot should be able to aggregate all the other robots around it. So we could predict that the emergence of leadership would be correlated to the distances from the barycenter of one robot with respect to the others. To calculate the “Barycenter Measure”, we have run a test where each robot (in turn) has been held in the centre of the environment, at a fixed position (motionless robot), while the other 3 robots have been allowed to move freely around the environment. The test has been repeated for 20 trials of 3000 time step each, and calculated over the last 20 generations At every time step, a distance between the motionless robot and the group’s barycenter has been calculated. First, we have calculated the barycenter coordinates according to the following formula:

$$x_{b}=\frac{\sum_{n}^{i=1}x_{i}}{n}\;y_{b}=\frac{\sum_{n}^{i=1}y_{i}}{n}$$ (1)

Where *x*_*b*_ and *y*_*b*_ are the barycenter’s coordinates and *x*_*i*_ and *y*_*i*_ are the coordinates of each robot. The distance between a robot and a group’s barycenter has been calculated by the “Euclidean Distance” formula:

$$d_{i}=\sqrt{{\left(x_{i}-x_{b}\right)}^{2}+{\left(y_{i}-y_{b}\right)}^{2}}$$ (2)

Where *x*_*b*_ and *x*_*b*_ are the barycenter’s coordinates, *x*_*i*_ and *y*_*i*_ are the coordinates of each robot, and finally *d*_*i*_ is the distance between the *i*–th robot and the group’s barycenter.

From the general averaging of time steps and trials, we derive a single value representing the extent of the robots’ “aggregation” all around the motionless robot. The final outcome is a sequence of 30 quadruples of values (one quadruple for each replication), where each value represents the distance between the “motionless robot” and the “group’s barycenter”. Since the “Barycenter Measure” is composed of quadruples of values, we have studied a summarizing measure called “Leadership Measure” which merges the information of each quadruple together into a single value. This resulting value is simply the standard deviation of each quadruple. The underlying idea is that the “quantity of leadership” in each replication might be the gap between the minimum distance (leader distance) from the group’s barycenter and the average distance of all the other robots (followers distance) since the stronger the influence of leadership on the followers, the shorter the distance will be to the groups barycenter.

### Who is the Leader?

Another measure has been devised to provide a general indicator of each robot’s individual capabilities: the “Individual Fitness Measure”. The Individual Fitness Measure is a “virtual” fitness function, since it has not been adopted for evaluating the robots during the evolutionary process, where the individual fitness depends on the collective behaviour of the group. Instead an individual virtual fitness might indicate the robot’s individual ability to reach the food zone. In order to weigh the individual fitness, we have designed a test where all the robots move freely in the environment, just as they do throughout the evolution. Every time a single robot reaches the food zone, the robot’s fitness score has been sampled and a counter increased independently from other robots’ fitnesses or from the global group’s fitness. Again, the test has been repeated for 20 trials and calculated over the last 20 generations. Thus, each fitness value represents the average over the trials and generations. Eventually, a single value has been obtained for each robot (for each replication), so producing a set of 30 quadruples.

### Is the Leadership a winning strategy?

Averaging the last 20 generations of the group fitness used for the HGA, we have determined a “Collective Fitness Indicator”. In our opinion, if a correlation can be observed between the “Leadership Measure” and this “Collective Fitness Indicator”, it means that the replications with a higher leadership also reach a higher fitness, meaning leadership is a winning strategy for the evolution of coordination.

### Types of Leadership

Another central issue concerning the leadership is whether leaders may opt for different behaviours depending on the “capabilities” of the followers. Leaders could adopt a form of active leadership whenever followers have a sub-optimal behaviour. The first measure, the “Capability of Followers”, evaluates the followers’ reaction times. After identifying the leaders of each replication (by means of the barycenters measure described previously), we have run a test where the leader (of each replication) has been made motionless and has been placed in a fixed position of the environment. Then we have measured the number of time steps that followers need before reaching their leader, on each trial. Each test has been repeated for 20 trials of 3000 cycles each, using only the best robots of the last generation. The reaction times have been averaged over the whole set of trials returning a single value for each replication. The second measure, called the “Mobility of Leaders”, evaluates the mobility of leaders of each replication. The hypothesis is that active leaders might be more mobile than passive leaders as they would continuously rearrange their trajectories according to scarcely reactive followers. Passive leaders could be characterised by the behaviour of walking towards the food zone ignoring the followers. The “Mobility of Leaders” has been calculated by counting the number of 5.5 cm x 5.5 cm sized cells (of the environment) which each robot visits during each trial. Every test has been performed on the last generation population of leaders for 20 trials lasting 3,000 time steps. Finally, a third measure characterising the leadership type assesses the vision system of the leader, that is the “Vision of Leaders”. This measure is grounded on the hypothesis that an active leader will tend to keep the followers in its visual field longer than a passive leader. Again we have run a test with all the robots acting in the environment, for 20 trials and 3000 time steps. For this test, we have counted (for each robot) the number of time steps in which at least one retina photo-receptor is activated.

### Temporal dependencies

The last question that we were interested in is: what are the evolutionary dynamics of leadership? For this investigation, we have recalculated the “Barycenter Measure” and the “Individual Fitness Measure” throughout all the generations, sampling by a step of 10 generations. Thereby we have produced temporal curves plotting the 4 barycenter and the 4 individual fitness scores for the 4 robots. These two set of curves have been called respectively: the “Temporal Barycenter Measure Curve” and the “Temporal Individual Fitness Curve”. To better manipulate the information about the distances in one single data, we have averaged the distance among the 4 barycenter curves. Eventually we averaged the gap between all the possible pairings of the 4 curves for each replication. The result is a plot of the “Temporal Distance Among Barycenter Measures” which we have overlapped with the “Temporal Collective Evolutionary Fitness” curve over the generations, for each replication. Later on, we produced a chart merging the “Temporal Individual Fitness Curve” and the “Temporal Barycenter Measure Curve” of the Leaders together. Both the first plot and the second plot have been smoothed by using Friedman’s Super-smoother. In order to highlight the temporal dynamics, we have calculated the derivatives of these curves. These derivatives provide us with the information about the points of curves with a maximum slope. The purpose of the first couple of overlapped curves is understanding whether the group coordination occurs in the moment a leader emerges, namely fitness (thus coordination) increases when a leader comes out (according to barycenters) The second couple of curves (overlapped) allow us to comprehend whether the best robots are the leaders, and what comes first: skills of the robot or leadership status?

## Results

### Behavioural Analysis

**Fig 4:**
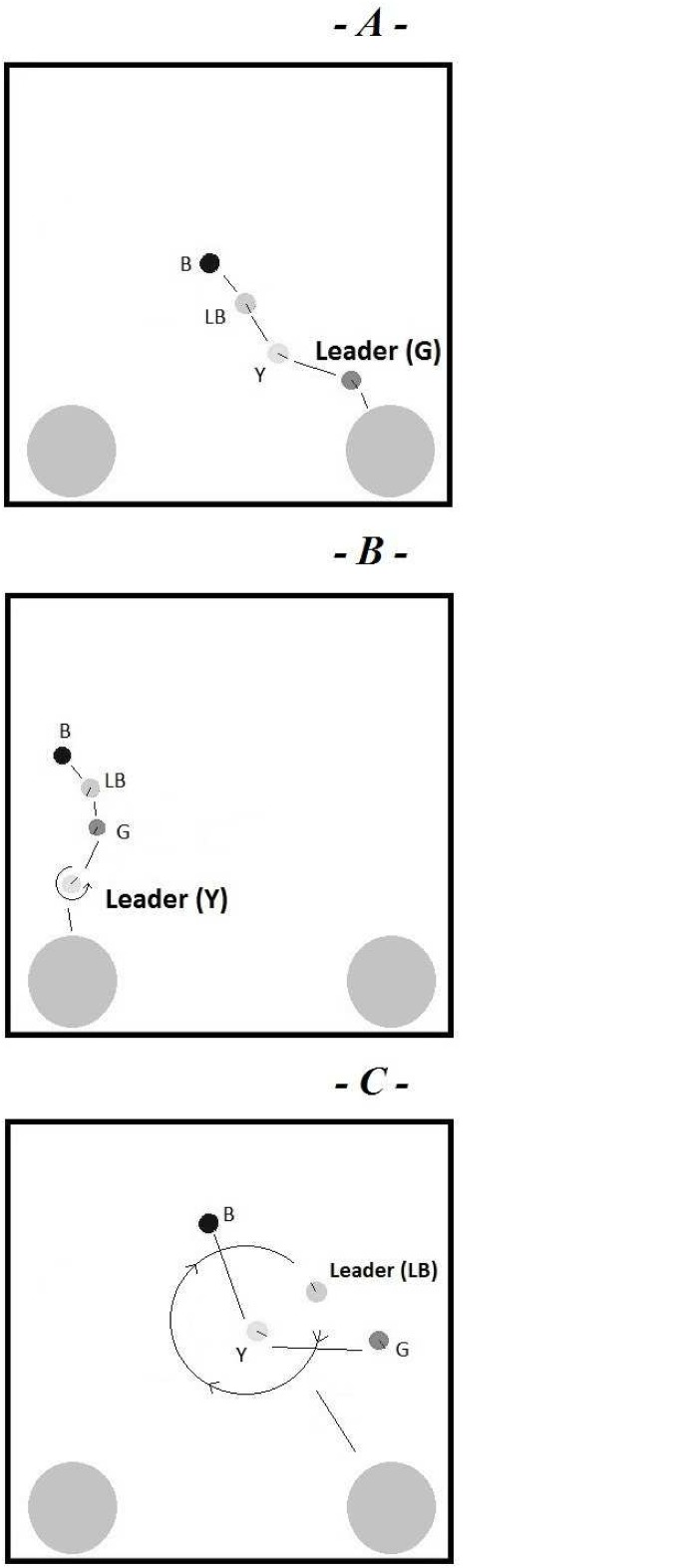
a) Passive Leadership. b) Weak Active Leadership. c) Strong Active Leadership.

Once the evolutionary process is terminated we have examined the behaviours exhibited by all best individuals at generation n. 1 (first generation), at intermediate generations, and at generation n. 600 (last generation). The first generation’s best robots appear “naive” and unable to make any meaningful actions. After a few generations, the robots start showing an effective exploratory behaviour that allows them to occasionally locate the food zones. Only after hundreds of generations, the robots exhibit a specific flocking behaviour [41] and are capable to carry out their adaptive task. Once it has evolved, such a flock remains stable up to the last generation. A more accurate look at the flocking reveals that, in every replication, it seems to be only one robot leading the whole group towards a target area, whereas the other robots tend to follow it. We will call the leading robot the “leader”, and the other robots the “followers”. The general dynamics of the group behaviour are: (1) at the beginning of each lifetime, the leader seems to choose between the two food zones under its own initiative and moves towards the selected food zone; (2) the leader’s movements affect all the followers actions, as they tend to follow the leader towards the selected food zone. In addition, across different replications we have observed different strategies of leadership: active leadership and passive leadership. In particular we could identify 3 main categories:

1. Passive Leadership: In this case, at the outset of its lifetime, the leader moves straight towards one of the two food zones. The leader always moves forward ignoring the followers. Moreover, the leader seems not to be affected by the followers behaviour. On the other hand, followers react to the sight of a leader by following it. This social system is strongly leader-centered as one individual leads and another follow. This situation is depicted in Fig 4a.
2. Weak Active Leadership: In this condition, the leader mainly moves straightforward the chosen food zone and the followers follow it as in the passive leadership situation. This situation is illustrated in Fig 4b. The main difference is that occasionally, the leader will suddenly turn on the spot.
3. Strong Active Leadership: In this situation leader looks very mobile and its trajectories tend to be circular, rather than linear, as in the previous cases. This condition is reported in the Fig 4c. Although the leader moves circularly, it will get closer the chosen food zone by slowly transferring the center of rotation.

### Numerical Analysis

**Fig 5:**
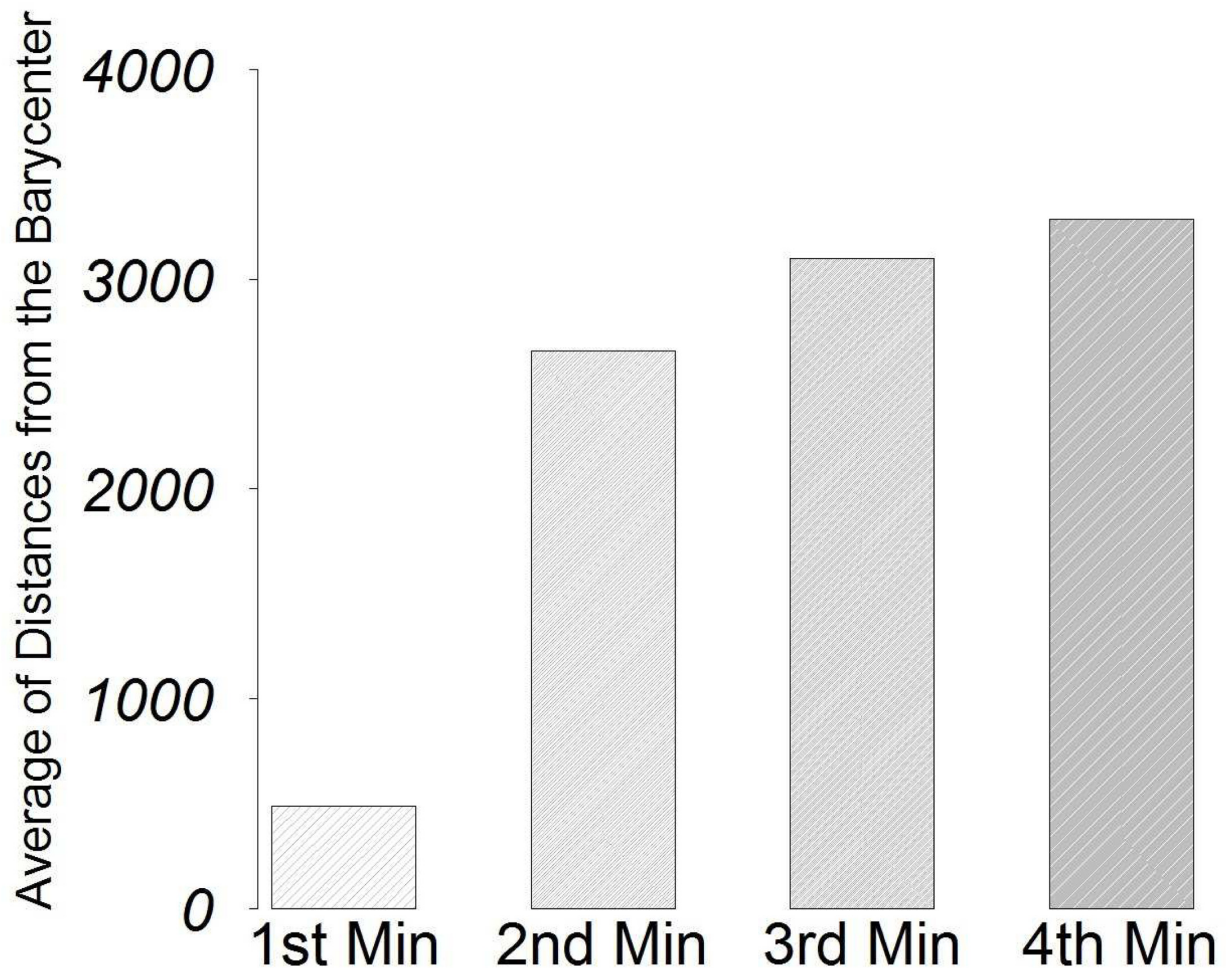
Representation of the average “Barycenter Measure” gap between the minimum and other robots.

Numerical analysis (already described in Section 3) has been applied to better investigate many of the behavioural observations presented in the previous section. The “Barycenter Measure” indicates that one of the four robots has always a minimum barycenter value with respect to other robots in the group. The bar-plot in Fig 5 depicts four averages, calculated over the 30 quadruples of the “Barycenter Measure”. In this chart, it is possible to notice a significant “gap” between the minimum average and the averages of the other three values, for each replication’s quadruple. This indicates that, in every replication, one of the robots in the group consistently turns out with the minimum distance from the group’s barycenter, which is an indication of leadership, as it means that the rest of group tend to remain around it.

**Fig 6:**
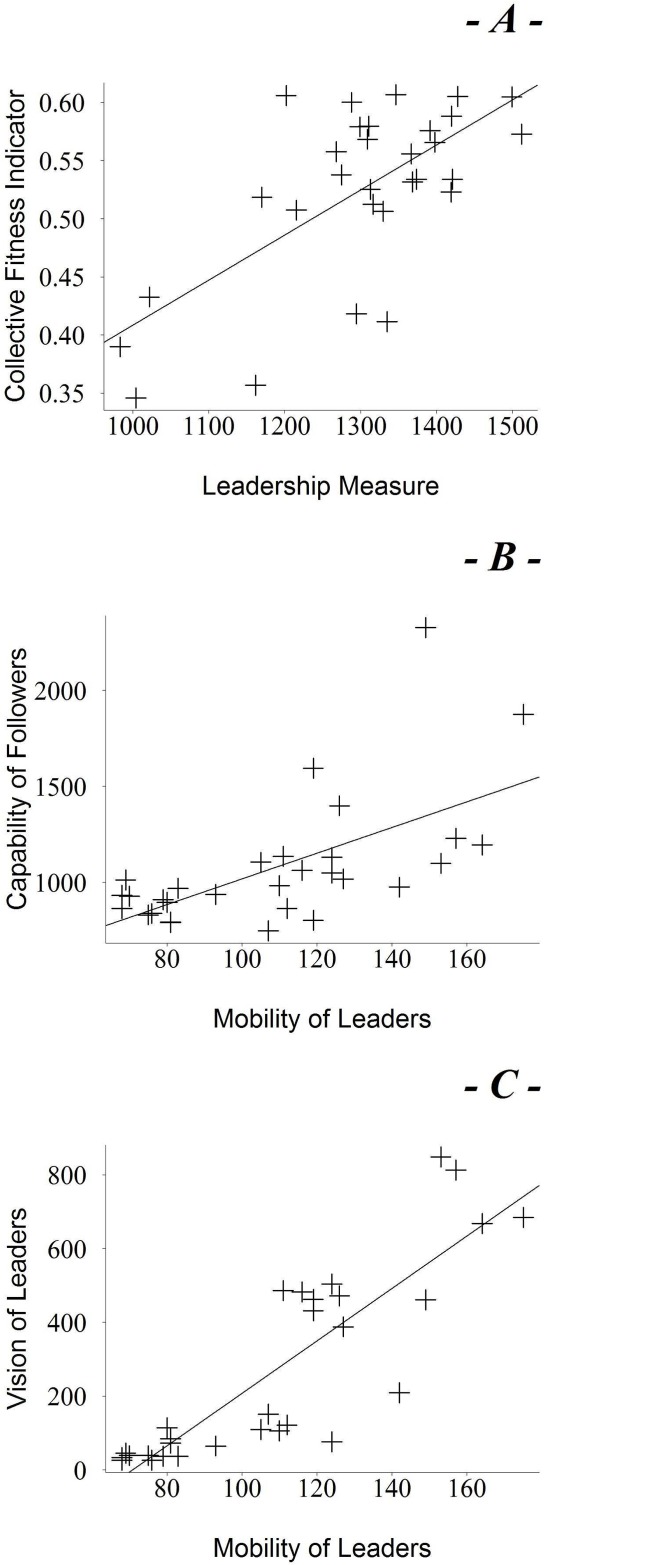
a) Correlation plot between the “Leadership Measure (standard deviation)” and the “Collective Fitness Indicator” over the replications. b) Correlation plot between the “Mobility of Leaders” and the “Capability of Followers” for each replication. c) Correlation plot between “Mobility of Leaders” and “Vision of Leaders”.

A comparison with behavioural observations has revealed that the observable leader is, in every replication (100% of replications), the robot with the minimum distance indicated by the “Barycenter Measure” (data not showed). Moreover, by comparing each “Barycenter Measure” quadruple with the “Individual Fitness Measure”, we can observe that every time a robot becomes the leader (i.e. barycenter value is the minimum) the same robot has the maximum individual fitness. This happens in the 100% of replications. This indicates that each leader, as defined above, is always the most skilled robot. As we stated in the numerical analysis description we need to correlate all the leaders information (over all the replications—“Barycenter Measure”) with the fitness (over the replications) in order to explain whether leadership is a successful strategy or not. The correlation between “Leadership Measure” and “Collective Fitness Indicator”, for each replication, is significant (Fig 6a, Pearson’s *ρ* = 0.67, p-value = 0.007928). Leadership appears as a winning strategy as every time there is a stronger leadership, there is also a higher fitness and vice-versa. The correlation between the “Capability of Followers” and the “Mobility of Leaders” is also significant (Fig 6b, Pearson’s coefficient with *ρ* = 0.62, p-value = 0.00005). This fact indicates that mobility of leaders correlates with the reaction times of followers. That is, the slower the followers, the more mobile the leader. This may indicate that leaders develop motility for optimising the group cohesion when followers are not able to follow and leaders become more mobile in order to affect the followers’ movements. Therefore, leaders’ mobility appears as a pre-requisite for the active leadership strategies. Finally, the correlation between “Mobility of Leaders” and “Vision of Leaders”, is also significant (Fig 6c, Pearson’s coefficient with *ρ* = 0.86, p-value = 0.0000000002). This result strengthens the hypothesis about active leadership: active leaders are more likely to be in constant motion and keep the behaviour of followers under constant control to optimise the social coordination.

**Fig 7:**
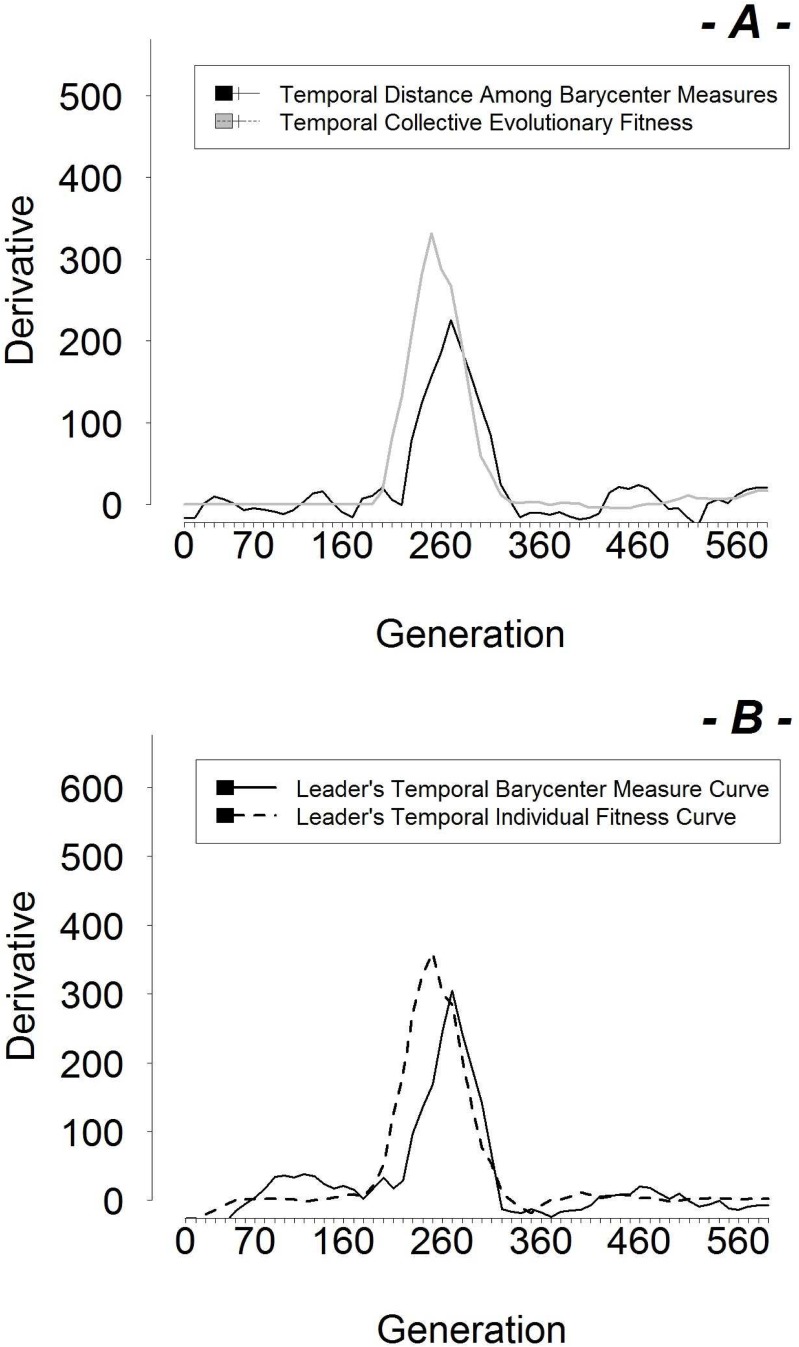
a) Comparison Charts of “Temporal Distance Among Barycenter Measures” and “Temporal Collective Evolutionary Fitness” derivatives related to the replication no.13. b) Comparison Charts of Learder’s “Temporal Individual Fitness Curve” and “Temporal Barycenter Measure Curve” derivatives related to the replication no.13.

This “active” strategy arises when in the group there are slow followers (this has been already proven by the correlation between “Mobility of Leaders” and “Capability of Followers”, see Fig 6b). This is in a great agreement with literature that (as we state in the Introduction and Conclusion sections) describes passive leadership arising in more homogeneous groups, in terms of behaviour, temperaments of members, etc. Active leadership arises in more heterogeneous groups. Now, the inner genetic differentiation (in reality and in our simulations) can produce “bad” followers (follower robots less capable to follow the leader or less reactive) in some replications. The presence of “bad” followers (namely a more heterogeneity or variance in robots behaviours) in some replications, forces the leader to assume a more active role with the group in order to improve the group cohesion and so maximising the fitness. So this inefficiency of followers generates an evolutionary pressure on leaders to develop an active leadership. Ultimately, these results might prove how active/passive leadership work in real life in the cases observed in literature. The charts of the “Temporal Analysis” are reported in Fig 7. The first part (see Fig 7a) is a comparison between the derivative curves relating to the “Temporal Distance Among Barycenter Measures” (black) and “Temporal Collective Evolutionary Fitness” (grey) curves over the generations, for one representative replication (no.13). As we can observe, there is an absolute maximum for each derivative curve which corresponds to the points of maximum slope inclination of the original curves. This chart shows that the curve of distance among barycenter measures grows at the same time as the group fitness curve. This fact indicates that when a leader emerges (that is when the distance among barycenter measures increases) then the group achieves a higher collective fitness which means the group starts to coordinate reaching the food zone. The second part of the picture (see Fig 7b) illustrates the derivatives of the “Temporal Individual Fitness Curve” curve (thick) and the “Temporal Barycenter Measure Curve” curve (thin) relating to the leaders of the replication no.13. As the barycenter has a decreasing trend, we have plotted the inverse of it in order to make it easy to compare both curves.

**Fig 8:**
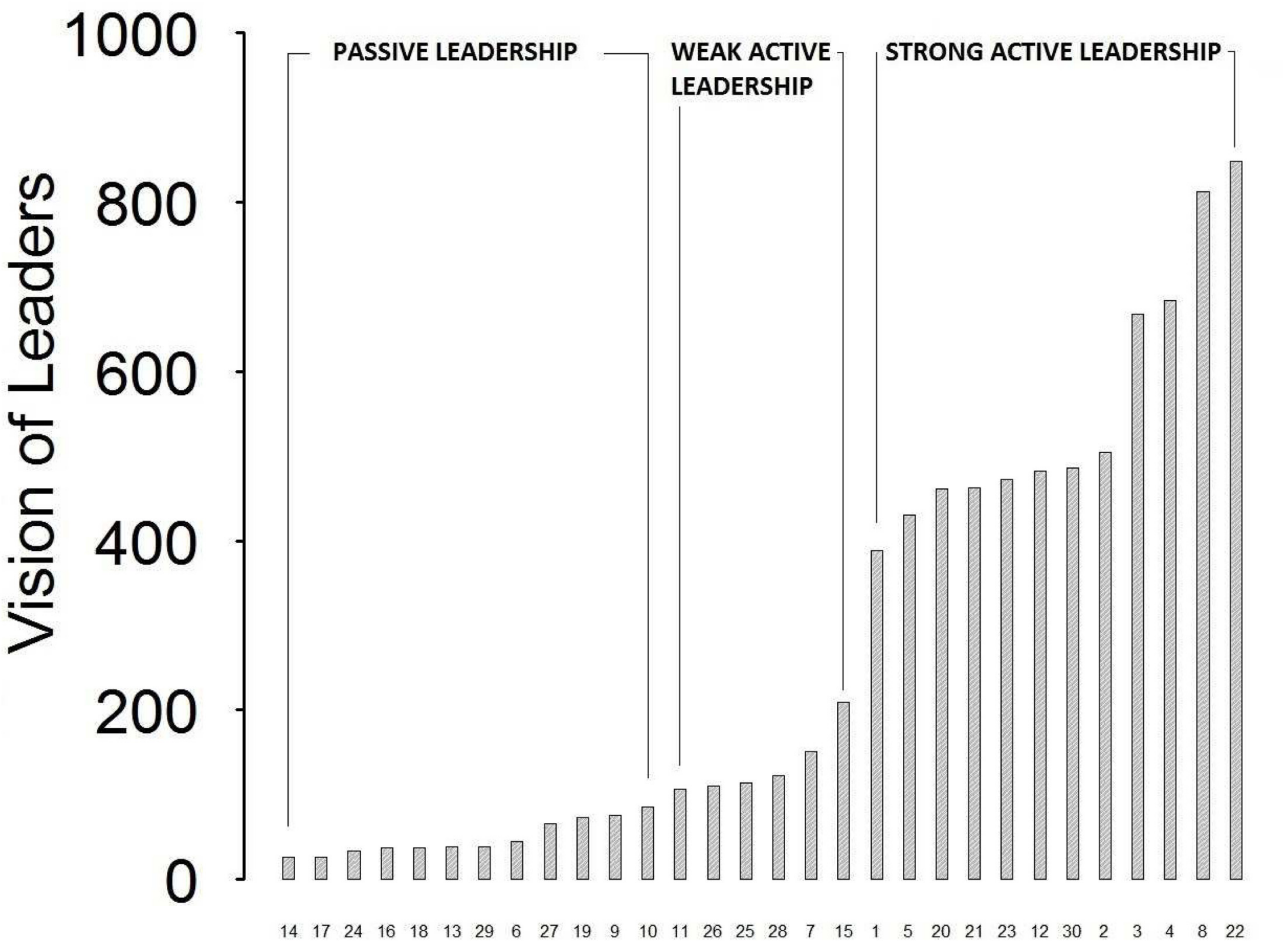
Sorted bar-plot of the leaders’ vision activations distinguishing between passive leaders, weak active leaders and strong active leaders.

We can notice that the maximum point of the individual fitness derivative comes prior to the maximum point of the barycenter derivative during the evolutionary process. This suggests that the robots’ skills evolve before the leadership status and right these better skills cause the emergence of the leadership. In other words, the individual fitness of the leader starts to grow when the robot becomes able to reach the food zone faster than the others. Only a few generations later, the robot starts to be the group’s leader (according to the barycenter measure): a robot can only become first the best (for the task) and consequently the leader of the group. We have observed that in 73% of replications, the maximum individual fitness curve derivative comes before the maximum barycenter derivative. This strengthens our hypothesis that leaders are robots able to reach the food zones first. In this way they become a behavioural attractor which draws the other robots towards them. This suggests that leadership is a consequence of the genetic traits of a robot. Finally, by looking at the strategy of leadership, the graph in Fig 8 shows the values of vision activations (“Vision of Leaders”) organised from the highest to the smallest recorded value. Interestingly, we have found that “Vision of Leaders” is in excellent agreement with the behavioural observation: whenever a specific leadership strategy is detected in one replication it matches with the related numerical indicator of vision. In particular, from replication no.14 to replication no.10 (left-hand side of the graph), leaders move straight forward, never turning or changing the direction of motors: these are the passive leaders. From replication no.11 to no.15 (center of the chart), leaders move straight most of the time and turn occasionally: these are the weak active leaders. Finally, from replication no.1 to replication no. 22 (right side), leaders describe circular trajectories and are characterised by high mobility: these are the strong active leaders. Over all the 30 replications, the distribution of the leadership strategies is as follows: passive leadership arose in around 50% of them in our simulation, the other 50% is active leadership consisting of a 30% of weak active leadership and 70% of strong active leadership.

## Conclusions

Our results illustrate that in a group of robots, heterogeneous leadership can evolve (S1 Video). In particular, the first correlation of our simulation analysis (“Collective Fitness Indicator” with “Leadership Measure”) suggests that the stronger the leadership, the higher the overall fitness of the group (which means higher level of group coordination). This makes leadership a winning strategy in group decision making problems. Interestingly, we noticed that the robot which emerges as a leader is also the best in reaching the food zone and foraging on it. This fact strengthens the empirical data and observations in biological literature [42, 43]. By looking at evolutionary dynamics, one can infer a possible explanation of the mechanisms underlying the emergence of leadership. When a group of robots starts to evolve, members are naive”. The group tends to remain spatially compact and distances from the group’s barycenter of each member are similar. However, after some generations, one robot continues to react better to the stimuli from the food zone, becoming more and more capable of reaching the food first. The predominant factors fostering the emergence of robots food-seeking abilities are mainly due to a suitable combination of the genetic traits. A secondary factor could be a favourable initial random position of the robot within the environment over the generations, as a closer initial location would make the robot more able to reach the food zone first. Thereby, the fastest robots will reach the food zone first in most of the generations. On the other hand, the slower robots evolve themselves in an environment where they mostly watch the best robot reaching a food zone before them. In this situation, the colour of the best robot becomes a behavioural attractor for the other robots during the evolution. The slower robots become followers since they learn how to react to the stimulus from the food zone sensor plus the visual stimulus from the specific robot that arrives first in the food zone more often. In other words, leaders evolve the ability to reach the food areas first and followers evolve the capacity to react to the leader’s colour. The outcome is that followers follow the leader everywhere within the environment. Furthermore, the leadership strategy appears the simplest and the most successful way for the group to coordinate, as leadership evolves in all the replications. More in detail, we can observe the emergence of different “styles” of leadership. In the case of “passive” leadership (S2 Video), the strategy is strongly based on the “capability” of followers. If followers are all similarly able to react to the leader movements and to follow them, the group cohesion is constantly kept higher by the effective follower’s behaviour. The leader will have no “concern” other than just heading to the food zone. It means that followers are homogeneous in their behaviour, as they tend to behave in the same way and they are all able to follow the leader in a similar fashion. This is in excellent agreement with literature that document the emergence of passive leadership in homogeneous groups of insects [33]. In other situations, leaders show instead forms of “active” leadership when followers are more heterogeneous in personalities (behaviours). In a “weak” form of active leadership (S3 Video), leaders slow down facilitating followers’ reactions to their movement. The leaders then quickly regain their original direction in order to pursue the objective of reaching the food zone. In the “strong” form (S4 Video), the leader traces circular trajectories in order to be in the middle of the group. This active way of moving by the leader improves the followers sight capabilities by ensuring that they can see the leader all the time. This improves the followers capacity to follow and increases group cohesion. The strong form of active leadership arises when the initial genetic and environmental factors determine a condition of followers with insufficient ability [44]. Consequently, the evolutionary process shapes pro-active leaders who “care about keeping the entire group compact and leading” it to the target area. In Table 1 we sum up a table with pros and cons of the different types of leadership merging insights from our breakthroughs and literature. During the test stage we performed many attempts to disrupt the original experimental setup status used during the evolutionary stage, in order to test the robustness of the evolved strategies. In those tests we removed one or more individuals from a group of evolved leaders-followers. At first we removed one follower at random. In a group of passive leadership this operation did not make any difference, all the group moved towards the food zones anyway. In a group of active leadership the leader kept looking for the vanished follower constantly without pursuing the reaching of the food zone. This shows that the leader behaviour is strongly influenced by the followers, in this case. Instead, by removing the leader, we observed that the group reached a deadlock as it could not coordinate anymore. This occurred both in active and in passive leadership, followers kept turning around themselves with no destination. So this proves that the whole evolved group is unable to coordinate without the leader. All these cases are clearly illustrated by videos in the “Supporting Information”.

**Table 1:** Cost/Benefit Table for the three types of leadership arised during the experiments.

	Passive Leadership	Weak Active Leadership	Strong Active Leadership
When occurs	This social system is leader-centered. Passive leadership usually occurs when individuals possess pertinent information and are able to react to the leader movements very well, so not needing active communication from the leader.	Active leadership occurs when potential leaders explicitly signal their intention to other group members that can choose whether to follow or not. This leadership generally operates at a local scale, that is, between local neighbours.	The strong form of active leadership arises when the initial genetic factors determine a condition of bad followers. Consequently leader acts in pro-active way to keep the entire group cohesive. This leadership generally operates at a global scale (entire group).
Costs	Inefficiency of the group coordination in terms of reseources optimisation, as everything depends on the leader motion. Scarce adaptability of the group to new conditions.	This is an intermediate situation, leaders waste time to slow down to wait for followers. Later they regain their initial direction to reach the objective.	The group spends time and slows down to allow the leader to negotiate choices with all followers actively.
Benefits	No time needed for the negotiation of the leader role. Leader just leads and followers follow him. The global efficiency of the group coordination may be higher than the other forms of leadership in terms of time, speed, etc.	A better group coordination with the respect to the passive case if followers that are not good. However coordination is not as good as in the strong case.	The group coordination and cohesion is high even though the followers are not good. In this way the exploitation of the environment resources is optimised.
Species	Homogenous insect swarms where individuals have no significant conflict of interest. [33]	ants (Temnothorax albipennis) [36], migrating honeybee colonies [37], etc.	Ravens [44], Dolphins [34], Primates using vocal and visual signals [35], etc.

## Supporting Information

S1 Video The evolutionary process of Leadership. This clip shows how robots evolve throughout the generations. They become smarter and smarter until a leadership emerges within the group. Furthermore the video highlight that the leader is the fastest robot in reaching the target area.
(MP4)

S2 Video The Passive Leadership. This clip illustrates the underlying mechanisms of the passive leadership strategy.
(MP4)

S3 Video The Weak Active Leadership. This clip shows how the weak active leadership works. From time to time, the leader turns right around holding the followers in check.
(MP4)

S4 Video The Strong Active Leadership. This clip illustrates how the strong leadership works. Basically the leader takes all the followers under constant control. In this way the leader optimises the group cohesion.
(MP4)
